# A Comparative Study of A_2_SiF_6_ (A = Cs, K) Phosphor Host Matrices: Linear Combination of Atomic Orbital Hybrid Density Functional Theory Calculations

**DOI:** 10.3390/ma18092025

**Published:** 2025-04-29

**Authors:** Leonid L. Rusevich, Mikhail G. Brik, Denis Gryaznov, Alok M. Srivastava, Ilya D. Chervyakov, Guntars Zvejnieks, Dmitry Bocharov, Eugene A. Kotomin

**Affiliations:** 1 Institute of Solid State Physics, University of Latvia, 8 Kengaraga Str., LV-1063 Riga, Latvia; mikhail.brik@ut.eeilja.cervjakovs@cfi.lu.lv (I.D.C.); guntars.zvejnieks@cfi.lu.lv (G.Z.); jevgenijs.kotomins@cfi.lu.lv (E.A.K.); 2 School of Optoelectronic Engineering & CQUPT-BUL Innovation Institute, Chongqing University of Posts and Telecommunications, Chongqing 400065, China; 3 Centre of Excellence for Photoconversion, Vinča Institute of Nuclear Sciences—National Institute of the Republic of Serbia, University of Belgrade, 11000 Belgrade, Serbia; 4 Institute of Physics, University of Tartu, W. Ostwald Str. 1, 50411 Tartu, Estonia; 5 Academy of Romanian Scientists, 3 Ilfov, 050044 Bucharest, Romania; 6 Current Chemicals, 1099 Ivanhoe Road, Cleveland, OH 44110, USA; srivastaam@outlook.com; 7 Transport and Telecommunication Institute, Lauvas Str. 2, LV-1003 Riga, Latvia

**Keywords:** LED, phosphors, Cs_2_SiF_6_, K_2_SiF_6_, first-principle calculations, DFT, hybrid functionals, atomic and electronic structure, vibrational and elastic properties, Raman and IR spectra

## Abstract

Cesium hexafluorosilicate (Cs_2_SiF_6_, CSF) and potassium hexafluorosilicate (K_2_SiF_6_, KSF) compounds are suitable hosts for luminescent impurities. In this work, the results of first-principle calculations of the basic properties of both these compounds are discussed and compared with the available experimental and theoretical data. The simulations were performed using the CRYSTAL23 computer code within the linear combination of atomic orbitals (LCAO) method of the density functional theory (DFT) and the advanced hybrid DFT-HF exchange-correlation B1WC functional. A comparative study of the structural, electronic, and elastic properties of the two materials is presented, along with a study of the dependence of properties on external pressure in the range of 0–20 GPa. In particular, the electronic properties with an emphasis on the effective atomic charges (by means of Mulliken analysis) and the chemical bonding properties (by means of crystal orbital overlap population (COOP) analysis) were addressed, with regards to the pressure effects. The structure of the valence bands at 0 and 20 GPa was compared. The vibrational properties of CSF and KSF were calculated, including the simulation of the one-phonon IR and Raman spectra. The calculated Raman spectra exhibit excellent agreement with the experimental ones. The pressure dependences of sound speeds and the Debye temperature are evaluated.

## 1. Introduction

Red-emitting crystalline phosphors are materials that produce bright red light upon excitation by an external energy source, such as ultraviolet or blue radiation. Hexafluorosilicate compounds, A_2_SiF_6_ (where A = K, Cs), are among the potentially attractive phosphor materials for lighting and display applications, and for generating red photons in phosphor-converted white light emitting diodes (pc-LEDs) [1,2,3].

The Mn^4+^ ion is the most common activator in A_2_SiF_6_ phosphors, and its electron transitions within the *d*-orbital configuration produce the characteristic bright red luminescence [4]. The narrow-band emission in the red spectral region makes these materials well-suited for generating warm white light in LED light sources [2]. The experimental data also indicate that these materials can achieve light emission efficiency up to 120 lm/W and a color rendering index of up to 80% [5]. Therefore, there is ongoing research to identify fluoride host matrices and develop surface passivation procedures to satisfy some of the key phosphor requirements, such as high thermal stability and resistance to hydrolysis [6]. The literature data also indicates that external pressure can significantly affect the luminescence of phosphors [7].

In this study, two alkali metal A-ions of different masses were chosen—namely, the relatively heavy cesium (Cs) ion and the much lighter potassium (K) ion—to compare their electronic and elastic properties and their pressure dependence. Special attention was placed on examining the electronic and vibrational properties.

Exploring the properties of luminescent materials under varying pressure conditions is a powerful approach to advancing their fundamental understanding and technological applications [8]. Pressure alters the crystalline environment surrounding the luminescent ion, leading to changes in both its optical and electronic properties. Furthermore, high pressure can induce new stoichiometries and crystal structures with unique electronic properties. By leveraging first-principle density functional theory calculations, researchers can investigate the impact of pressure-induced transformations on the crystal structure, as well as on the elastic, mechanical, optical, and electronic properties of these materials. These investigations are particularly meaningful when directed at materials currently in commercial use. Fundamental research has the potential to accelerate advancements in material performance and pave the way for the discovery of new materials with significant commercial value.

To date, a number of first-principle DFT calculations for A_2_SiF_6_ systems have been performed to study the structural, electronic, optical, and mechanical properties under varying conditions [9,10,11,12,13,14,15]. Plane wave VASP and CASTEP computer codes, along with PBE, SCAN, and HSE06 functionals, were applied to model the crystalline structures, electronic states, and pressure-dependent behavior. The calculations confirmed the structural instability of NaKSiF_6_, and reproduced optical transition energies [9]. For Rb_2_MF_6_ (M = Si, Ni, Pd) and Cs_2_XF_6_ (X = Si, Ge), simulations highlighted lattice compression, nonlinear bandgap changes, and elastic property variations under pressure [11,12]. Doping with Mn^4^⁺ in K_2_SiF_6_ increased the dielectric constant and optical absorption range while decreasing the bulk modulus [10,14]. Recently, we have performed calculations of the basic properties of KSF under external pressure and without pressure using the LCAO method [15]. Such a study had not been reported in the literature. In this previous work, we provided the first comprehensive analysis of the vibrational properties of KSF. Several hybrid DFT functionals were employed, providing a more accurate description of the structural and electronic properties compared to the LDA functional. In particular, the bandgap value is ~10 eV if calculated by the hybrid DFT functional versus ~8 eV if calculated by the LDA functional. The best agreement between the calculated structural properties and the available experimental values from the literature was due to the hybrid B1WC functional.

In the current study, within the framework of the LCAO method, we present a comparative analysis of the basic properties of CSF and KSF using first-principle DFT calculations. The B1WC hybrid functional was employed, providing a more accurate description of the electronic structure compared to GGA or LDA calculations, which were used in most of the previous studies. The analysis includes projected density of states (PDOS) and crystal orbital overlap population (COOP) to explore bonding characteristics under pressure. This work complements previous studies, which primarily have focused on the effects of structural distortion, without specifically addressing trends in electron charge density redistribution. Additionally, we extend the pressure analysis to include elastic constants, Young’s modulus, and Poisson’s ratio, highlighting the pressure-driven changes in structural rigidity. Further, we present the results of our calculations and analysis of the vibrational properties, including simulated Raman and IR spectra. The calculated Raman spectra are compared with the experimental data.

## 2. Materials and Methods

**Figure 1:**
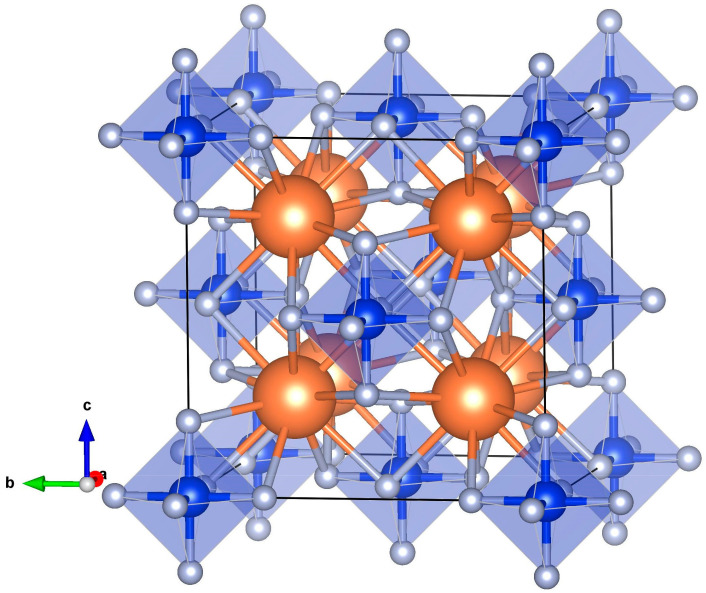
Sketch of the ideal crystal structure and crystallographic unit cell (36 atoms) of A_2_SiF_6_ (where A is Cs or K cation) [15]. A-ions are orange balls, Si—blue, F—grey. The unit cell is presented by a cube drawn with black lines.

Both the CSF and KSF crystals have the same face-centered cubic (fcc) crystal structure, characterized by the *Fm-3m* space group symmetry (Nr. 225). In this structure, the A-cations (either Cs or K) are surrounded by twelve fluorine anions, while the Si cations are coordinated by six fluorine anions, which create a perfect octahedron. The conventional (crystallographic) unit cell of this cubic lattice contains 36 atoms, which are in the following Wyckoff positions: A at 8c (1/4, 1/4, 1/4), Si at 4a (0, 0, 0), F at 24e (*x*, 0, 0). Figure 1 illustrates the ideal crystal structure of A_2_SiF_6_, highlighting the SiF_6_ octahedra and conventional cubic unit cell. Note that the SiF_6_ octahedra are situated at the corners and face centers of the cube.

The computational methods applied in our calculations are described in detail in our previous paper [15]. Here, we provide only a brief overview, focusing on key aspects and specific details relevant to the present calculations.

The first-principle (ab initio) computations of CSF and KSF crystals were performed within the LCAO method, as implemented in the CRYSTAL23 computer code [16,17]. We consider and compare here the calculated structural, electronic, elastic, vibrational, and dielectric properties of crystals with fully optimized geometry (with particular attention to the electronic properties). For some of the chosen properties, and for the Debye temperature, the dependences on external hydrostatic pressure (0–20 GPa) were studied, which allows us to estimate how changes in interatomic distances affect the basic properties of crystals. In this case, the optimized structure at each pressure was used for the calculations. Note that the CRYSTAL23 code, in addition to geometry optimization, also gives possibility to calculate the elastic properties of crystals under external pressure [17,18,19]. Additionally, the simulations of the one-phonon Raman and infrared (IR) spectra of crystals were performed.

In our previous study [15], we discussed and compared several basis sets and hybrid functionals for simulating the properties of the KSF crystal. Therefore, we have adopted the recommendation of this previous study to select appropriate methods for comparing CSF and KSF. The all-electron TZVP_2012 basis sets of Gaussian type functions were used for description of K, Si, and F atoms [15,20], while the TZVP_rev2 Gaussian basis set with pseudopotential was used to describe Cs atoms [21]. All these basis sets are available on the CRYSTAL23 Basis Sets Library web site [22]. The B1WC global hybrid exchange-correlation functional was employed for calculations. This single-parameter hybrid B1WC functional combines the Wu–Cohen WCGGA exchange functional with 16% of Hartree–Fock (HF) exchange and the Perdew–Wang PWGGA correlation functional [17,23]. It should be noted that the B1WC functional and the TZVP_2012 basis sets were previously chosen as optimal combination for the KSF simulation [15].

After geometry optimization, a vibrational analysis was performed for the considered systems. The frequencies of the transverse optical (TO) vibrational modes and vibrational contribution to the dielectric tensor were calculated at the Γ-point (in the center of the first Brillouin zone) within the harmonic approximation. Note that the complex dielectric function *ε*(*ν*), depending on the frequency *ν* and vibrational modes, is the sum of the electronic (high-frequency) *ε_el_* and ionic (vibrational) *ε_vib_* components: *ε*(*ν*) = *ε_el_* + *ε_vib_*(*ν*) [15]. CRYSTAL23 allows us to calculate both of these components, as well as the static dielectric tensor (static dielectric constant, in our case) *ε*(0) with an estimate of both the electronic and ionic contributions. The integrated intensities for the IR- and Raman-active vibrational modes were calculated. The one-phonon Raman and IR absorbance spectra, generated by the corresponding TO vibrational modes, were also simulated (see the details in Ref. [15]).

## 3. Results and Discussion

### 3.1. Selected Structural and Electronic Parameters

The basic CSF and KSF parameters calculated for fully relaxed systems are summarized in Table 1. This table includes the following data: lattice constant *a*, non-dimensional free *x* coordinate of the F ion (Wyckoff position 24e), distances between ions (Si–F, A–F), band gap *E_g_*, Mulliken (effective atomic) charges of ions, and refractive index *n*, determined as the square root of the electronic (high-frequency) dielectric constant. Experimental values (if available) of the corresponding parameters are provided in parentheses.

As can be seen from Table 1, the calculated structural parameters of CSF are in excellent agreement with the available experimental data. The relative difference in the lattice constant values is only −0.27%, while in the lengths of the Si–F bond it is 0.41%. For the KSF crystal, the discrepancies between the calculated and experimental structural parameters are as follows: −0.59% (or 0.50% compared to the low-temperature data from Ref. [24]) for the lattice constant, 0.89% for the Si–F distance, and −0.69% for the K–F distance. Thus, the errors in calculations of the mentioned structural parameters are less than 0.9% for both compounds. The achieved accuracy is significantly better than that of plane wave calculations using the LDA and GGA functionals [10,12,13,26,27], and is comparable to plane wave calculations with PBE0 and HSE06 hybrid functionals [27].

The values of *x* parameters, exhibited in Table 1, are free coordinates of the F ions (Wyckoff position 24e) in the CSF and KSF crystals, obtained after full geometry optimization. For the KSF crystal, the calculated value of this parameter coincides very well (0.24%) with experimental one measured at low temperature (110 K) [24].

Our calculations suggest 10.39 eV for the indirect band gap for the CSF crystal. However, the direct band gap at the Γ-point differ from this value only by 0.03 meV. In contrast to this, the calculated band gap for KSF is direct, and its value is a little smaller, 9.73 eV. This band gap is very close to that found for KSF with the HSE06 hybrid functional and plane wave calculations [27]. In general, calculations with hybrid functionals predict significantly larger band gap values than LDA and GGA functionals [10,12,13,26,27,28], which we have already discussed in Ref. [15]. Keeping in mind that LDA and GGA calculations tend to underestimate the band gaps, the results obtained in our calculations seem quite reasonable. Since no reliable experimental data on the CSF and KSF band gaps were found, it is unfortunately not possible to directly compare the calculated band gaps with the experiment.

The Mulliken effective charges of Cs and K ions in Table 1 confirm the ionic nature of the A-site ions, as these charges are close to the formal charge of +1|e|. These ions donate about 90% of the charge of the valence electron to form a bond. Nevertheless, *q*(Cs) is a bit larger in the comparison with *q*(K), which leads to the difference between the two materials reflected in the calculated electronic properties (see Section 3.2 and Section 3.3 below). The calculated Mulliken charge of Si ions in both materials is about +1.8|e|, which is ~45% of the formal ionic charge of Si^4+^ ion (formal oxidation state +4) and is related to partial covalence of the Si–F bonds. Each F ion acquires a charge of about −0.6|e| in both materials. It is obvious that, for both compounds, the degree of ionicity for the A–F bond is much higher than for the Si–F bond. The presented analysis of the calculated ionic charges is in line with the values of the electronegativity of the elements. The most widely used is the thermochemical Pauling scale, where electronegativity has units of eV^1/2^. According to this scale, the electronegativity of the elements included in CSF and KSF is X(Cs) = 0.79, X(K) = 0.82, X(Si) = 1.90 and X(F) = 3.98 [29,30]. Here the electronegativity of Cs and K is significantly different from that of fluorine; thus, they are supposed to form Cs–F and K–F ionic bonds, whereas Si has the electronegativity lying in-between. Additionally, let us use the simple heuristic formula proposed by Pauling [29,31,32] to estimate the degree of ionicity of the interatomic bond (or, originally called the partial ionic character of a single bond) between elements E_1_ and E2 as follows:

f(E_1_–E_2_) = 1 − exp{−0.25·[X(E_1_) − X(E_2_)]^2^}, (1)

where X(E_1_) and X(E_2_) are the electronegativities of the corresponding elements. Calculations using Equation (1) yield the following degree of ionicity of bonds: f(Cs–F) = 92.1%, f(K–F) = 91.8%, f(Si–F) = 66.1%. Overall, these results indicate that the character of the Cs–F and K–F bonds is almost entirely ionic, whereas the Si–F bonds contain about 34% covalency.

The experimental value of the refractive index *n* for CSF, which in Table 1 is given for *λ* = 589.3 nm, coincides very well with the calculated value. In general, the difference between the calculated and experimental values of the refractive indices for both compounds does not exceed 1.5% (see Table 1).

### 3.2. Effect of External Pressure and Elastic Properties

**Figure 2:**
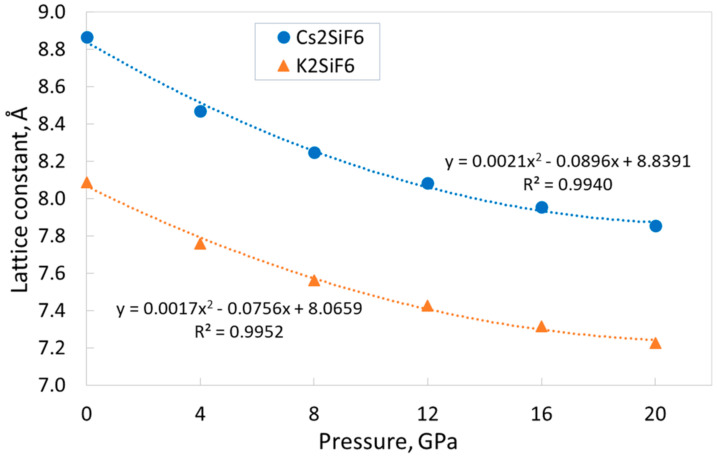
Dependences of the lattice constants for CSF and KSF on external pressure (filled markers) and the corresponding fitting (dotted lines).

To evaluate how the external hydrostatic pressure affects the main structural, electronic, and elastic properties of CSF and KSF, we performed calculations of the dependences of these parameters on the pressure in the wide range of 0–20 GPa. The graphs of the dependences of the lattice constants on pressure are presented in Figure 2. These dependences could be successfully approximated by the second-order polynomial functions, that allows for reliable values of the studied parameters in the entire range of the considered pressures. Figure 2 reveals that, as expected, the lattice constants decrease with increasing pressure, and these dependences are qualitatively similar for both materials. However, there is a slight difference. The CSF lattice constant decreases by 11.4% when the pressure increases from 0 to 20 GPa, whereas the KSF lattice constant decreases by only 10.6%. The somewhat smaller change of the KSF lattice constant under external pressure implies a slightly greater rigidity of its crystal structure (see below the discussion of the elastic properties of CSF and KSF). Note that a decrease in the lattice constant by ~11% leads to a decrease in the crystal volume by ~30%.

**Figure 3:**
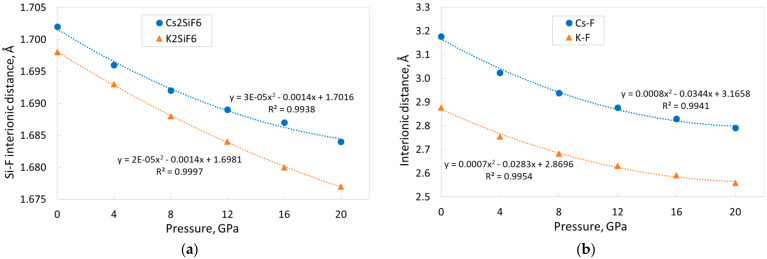
The pressure dependence of the interionic distances in the CSF and KSF crystals along with the corresponding fitting (dotted lines): (**a**) Si–F bonds; (**b**) Cs–F and K–F bonds.

Let us consider the interionic distances in CSF and KSF. As can be seen from Table 1, the Cs–F and K–F distances at ambient pressure are almost twice the Si–F distance. Figure 3 shows that all interionic distances, as well as the lattice constants, decrease monotonically with increasing pressure. Nevertheless, the rate of decrease of these parameters differs. We have already drawn attention in Ref. [15] to the fact that, in KSF, the rigidity of the Si–F bond is significantly higher than the rigidity of the K–F bond; the decrease of interionic distances at 20 GPa is 1.2% and 11.1% for the Si–F and K–F bonds, respectively. The same tendency also holds for the CSF crystal, in which, at the external pressure of 20 GPa, the Si–F distance decreases by 1.1%, and the Cs–F distance decreases by 12.1%. This reduction of interionic distances means that the volume of SiF_6_ octahedra decreases by ~3%, while the volume of the CsF_12_ polyhedra decreases by ~32%. Thus, it is essential that the decrease in the volume of the unit cell of CSF and KSF with pressure occurs mainly due to the decrease in the size of the CsF_12_ and KF_12_ polyhedra, respectively. The difference in rigidity of the Si–F and A–F bonds could be explained by the fact that the nature of the Si–F bonds, unlike the A–F bonds, includes a significant contribution of covalence (see the discussion in Section 3.1 and Section 3.3). If we compare the CSF and KSF structures, we can conclude that the rate of decrease of distances between the Cs and F ions in CSF is higher than between the K and F ions in KSF, while the rate of change of the Si–F distance is almost identical in both crystals. This fact could again indicate a slightly greater rigidity of the KSF crystal structure.

**Figure 4:**
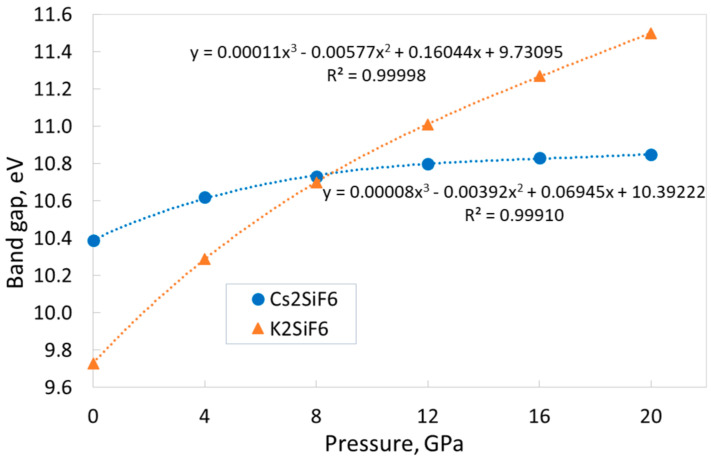
Band gap dependences of CSF and KSF on the external pressure and the corresponding fitting (dotted lines). Calculations give an indirect band gap for Cs_2_SiF_6_ and a direct band gap for K_2_SiF_6_ in the considered pressure range.

The calculations reveal that both the CSF and KSF materials are wide-gap insulators (see Table 1). At the same time, the band gap of both crystals increases monotonically with pressure, but the behavior of these dependences is different (Figure 4) in two materials. The KSF band gap increases by 18.2% in the pressure range of 0–20 GPa, showing only minimal signs of slowing growth. In contrast to this, the CSF band gap increases slowly by only 4.4%, and this dependence demonstrates a clear trend of slowing down. Both dependences are perfectly described by third-order polynomial functions (see Figure 4). Note that, over the entire pressure range, the calculations yield indirect and direct band gaps for CSF and KSF, respectively. At the same time, direct band gaps of CSF at various pressures differ from indirect ones at the same pressures by the minimum values (see Section 3.1).

Table 2 demonstrates the change of Mulliken charges of ions in CSF and KSF when the external pressure is applied. The calculated charges are in line with a high degree of ionicity, as previously discussed for zero pressure. That is, the alkali metal atoms exhibit charges close to the formal value of 1|e|, indicating the ionic character of the bonds. In both compounds, charges (in absolute value) of A-cation and fluorine decrease monotonically with increasing pressure. The charges of Cs and K change by 0.095|e| and 0.078|e|, respectively, while the charges of the F ions change by 0.034|e| in CSF and by 0.024|e| in KSF. This conclusion reveals a completely opposite tendency when compared with the results of plane wave calculations using the LDA and GGA functionals [12,13] for the Mulliken charges, which indicated the charges (in absolute value) of A- and F-ions increased with increasing pressure. The complex material response to the pressure does not allow us to conclude which of the two trends should take place. It is worth noting that the calculation of Mulliken charges using the LCAO basis set is a more natural combination. Considering that our present results and those reported in [12,13] regarding the pressure dependences of interionic distances and lattice parameters show qualitative similarity, we plan to incorporate the calculation of Bader charges for the plane wave calculations in a future study. At the same time, the changes in the charges of Si ions in both CSF and KSF do not exceed 0.01|e|; thus, to a first approximation, they can be considered more or less constant. In general, such behavior of ionic charges indicates that, with increasing pressure, the ionicity of bonds (at least the A–F bonds) slightly decreases.

**Figure 5:**
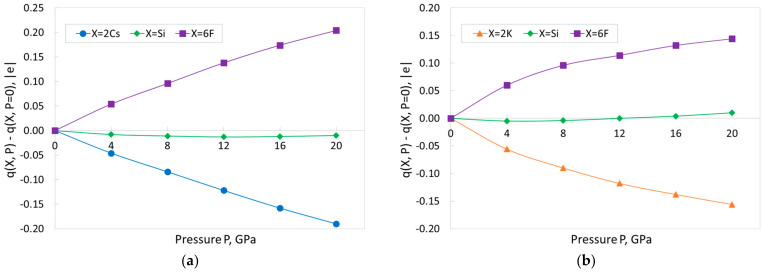
The pressure induced changes in the total charges of three types of ions (Cs or K, Si, F) forming the formula unit of the compounds relative to the ambient pressure: (**a**) CSF; (**b**) KSF. The lines are shown only as a guide to the eyes.

Figure 5 visualizes the above-mentioned trend. This figure shows the changes of the pressure dependence of the total charges of the three types of ions (Cs or K, Si, F) forming the formula unit for CSF (Figure 5a) and KSF (Figure 5b). The crystal formula units include the two Cs or K ions, one Si ion, and six F ions. Figure 5 reveals that the A-cations charges become less positive, the charges on the fluorine become less negative, and the charges on the silicon remain practically unchanged. Note that the sum of all charges at any pressure in Figure 5 is zero, to maintain the electric neutrality of the crystal lattices.

The pressure effect on certain CSF elastic properties is seen in Table 3. CSF has a cubic crystal system, so the symmetric elastic tensor of CSF has three independent non-zero components *C*_11_, *C*_12_, and *C*_44_. These elastic constants, along with bulk modulus (*B*), shear modulus (*G_H_*), Young’s modulus (*E_H_*), and Poisson’s ratio (*ν_H_*) in the Hill averaging scheme, are collected in Table 3. Note that the Hill moduli are obtained by averaging the Voigt (as an upper limit) and the Reuss (as a lower limit) respective moduli [33,34], and the Voigt, Reuss, and Hill bulk moduli of CSF (and KSF) are equal.

We begin by outlining some qualitative insights. Our calculations confirm that the necessary and sufficient Born stability criteria for the cubic crystalline systems [35]

*C*_11_ − *C*_12_ > 0, *C*_11_ + 2*C*_12_ > 0, *C*_44_ > 0 (2)

are fulfilled for the CSF crystal. The same conclusion applies to the KSF crystal, based on the evaluation of its elastic constants (see Ref. [15]). An analysis of Table 3 reveals that all elastic constants and moduli of CSF presented in the table increase with increasing pressure. The elastic properties of KSF demonstrate qualitatively the same behavior [15]. Moreover, both crystals, CSF and KSF, satisfy the condition *C*_12_ > *C*_44_ for all pressure values. This relation is typical for ionic structures; whereas, for covalent compounds, the opposite relation *C*_12_ < *C*_44_ is valid [12]. From this point of view, both crystals can predominately be classified as ionic compounds. A comparison between the calculated values of the elastic constants and the moduli of CSF (Table 3) and KSF (Table 4 in Ref. [15]) reveals that the values of all elastic quantities, with the exception of *ν_H_*, are larger for KSF. Thus, the calculation of elastic properties confirms that the KSF crystal is more rigid than the CSF crystal. This conclusion is also consistent with a well-established observation that, in cubic crystals, the values of elastic parameters increase as the lattice constant decreases.

Let us consider in more detail the Poisson ratio *ν_H_*. At zero external pressure, the *ν_H_* of KSF is slightly higher than that of CSF: 0.271 vs. 0.268. However, already, at 4 GPa, the situation turns out to be the opposite. With a further increase in external pressure, the *ν_H_* of CSF increases much faster than KSF and, at 20 GPa, the increase in the Poisson ratio of CSF is 58%, versus 33% for the Poisson ratio of KSF. To the best of the authors’ knowledge, this is the first study of the pressure dependences of the Poisson ratio for CSF and KSF, so it is not possible to compare the obtained results with other calculations.

Now, let us analyze other elastic parameters of CSF (Table 3) and compare them with the corresponding parameters of KSF. As previously mentioned, for all pressures, these elastic parameters for CSF are less than those of KSF. However, such a simple conclusion does not hold for the pressure dependent rate of growth of elastic parameters. When the pressure increases from 0 to 20 GPa, the elastic constant *C*_11_ for CSF increases by 287%, and for KSF by 310%; for *C*_12_, 721% (CSF) vs. 589% (KSF); for *C*_44_, 105% vs. 286%; for *B*, 474% vs. 435%; for *G_H_*, 68% vs. 208% and for *E_H_*, 88% vs. 229%. For both materials, the elastic constants *C*_12_ increase most rapidly, followed by the bulk moduli B. It is clear that, for the six considered parameters, two (*C*_12_ and *B*) increase faster in CSF. The situation with the elastic constant *C*_12_ is even more interesting. If, at 0 GPa, the value of this constant for CSF is smaller than for KSF (12.48 GPa vs. 14.94 GPa), then at 20 GPa they are already practically equal (102.40 GPa vs. 102.94 GPa).

The effect of isostatic external pressure on the elastic properties of KSF was considered in Ref. [10]. Three elastic constants, bulk and shear moduli were calculated at different pressures using the VASP plane-wave calculation package and GGA PBE functional. The qualitative conclusion in this paper is the same: all considered elastic parameters increase with increasing pressure.

### 3.3. Electronic Properties

**Figure 6:**
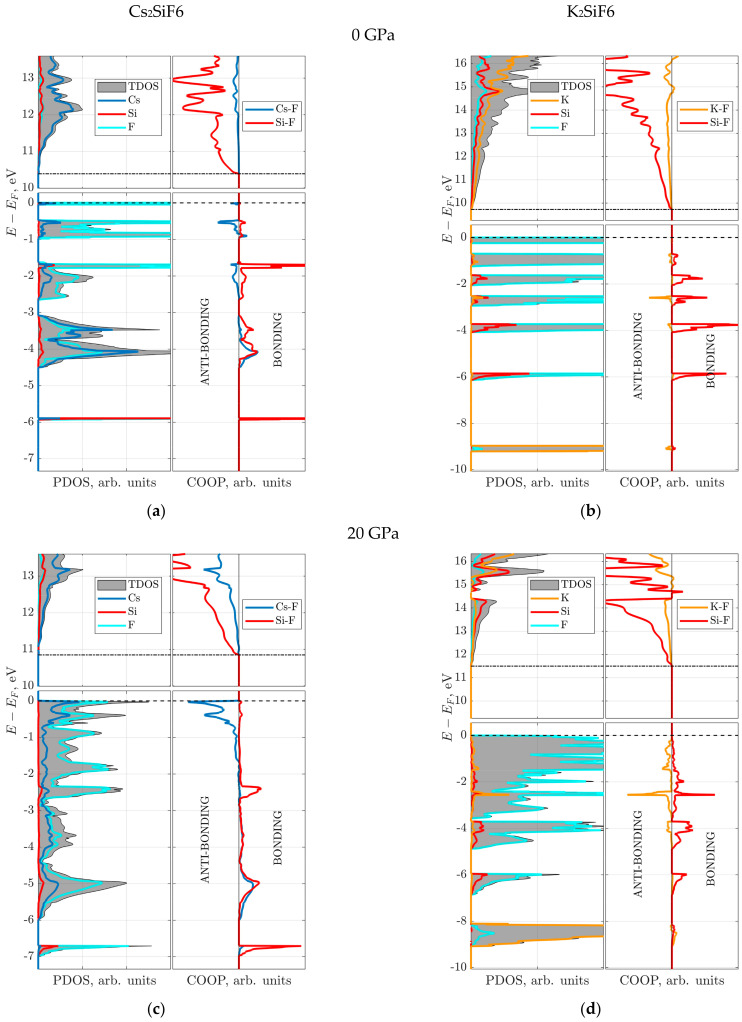
Atom projected density of states (PDOS) and crystal orbital overlap population (COOP) analysis for the CSF and KSF crystals in the cubic *Fm-3m* phase: (**a**) CSF at 0 GPa; (**b**) KSF at 0 GPa; (**c**) CSF at 20 GPa; (**d**) KSF at 20 GPa. Fermi levels and the bottom of conducting bands are marked with dashed and dot–dashed lines, respectively. Bonding (positive) and anti-bonding (negative) regions are indicated in the COOP plots.

Firstly, the electronic structure is analyzed using the atom projected density of states (PDOS) for pressures of 0 and 20 GPa (Figure 6). The bottom of the conduction band (CB), marked by the dot–dashed line, is formed mainly by A-cation (Cs or K) states. Deeper analysis of the CB bottom revealed an intermixed contribution from corresponding A-cation and Si *s*-states. For both crystals, the following common properties can be clearly seen from the calculated PDOS: (a) the valence band (VB) consists of several sub-bands, spanning the energy range between 0 and −6 eV; (b) the F-*p* states dominate the very top of the VB at 0 eV, marked by a dashed line (Fermi level). However, a qualitative difference between the two materials still exists in the properties of sub-bands at 0 GPa: (i) the states forming the top of the VB in CSF, in addition to the F-*p* states, include a contribution from the Cs-*p* states (Figure 6a,c), which becomes more pronounced in the deeper sub-bands; (ii) the Si contribution increases in the deeper sub-bands in KSF; (iii) smaller number of and broader sub-bands in CSF than in KSF; (iv) the deep sub-band at -6 eV is mainly due to the strong inter-mixture of Si and F states in CSF. Moreover, at 20 GPa for CSF, the Cs-*p* contribution increases considerably, almost reaching that of the F-*p* states (Figure 6c) in the energy range between 0 and −0.5 eV. For both crystals, expansion and partial merging of the sub-bands is observed at 20 GPa (Figure 6c,d).

**Figure 7:**
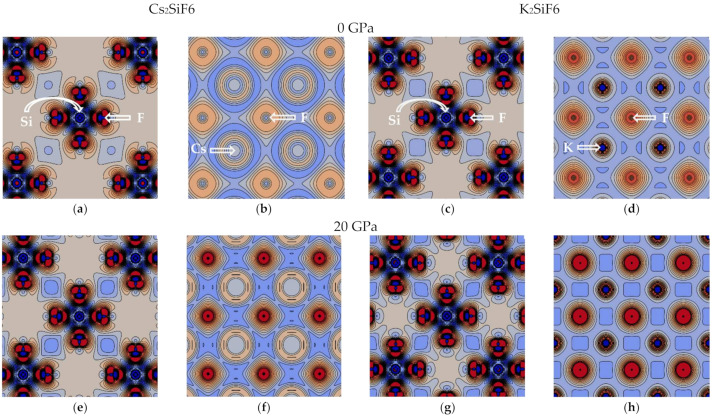
Charge density difference plots for the CSF and KSF crystals: (**a**,**b**) CSF at 0 GPa; (**c**,**d**) KSF at 0 GPa; (**e**,**f**) CSF at 20 GPa; (**g**,**h**) KSF at 20 GPa. We distinguish the Cs–F (**b**,**f**), K–F (**d**,**h**), and Si–F (**a**,**c**,**e**,**g**) planes. Blue and red depict electron density decrease (from −0.010 |e|/bohr^3^) and increase (up to 0.010 |e|/bohr^3^) with step size of 0.001 |e|/bohr^3^, respectively.

Secondly, to discuss the nature of the Si–F and A–F bonds, the electronic subsystem is analyzed using *crystalline orbital overlap population* (COOP) [17,36] and the electronic charge density difference (i.e., the difference between crystalline and superposition of non-interacting neutral atomic densities) plots (Figure 6 and Figure 7). The present COOP analysis is analogous to a more popular COHP (crystalline orbital Hamilton population) analysis for the plane wave calculations [37]. As mentioned above for the atomic charges, the methods requiring local basis are less suitable for the plane wave calculations. The COOP calculated from the overlap matrix and coefficients of linear combination of atomic orbitals is natural for the LCAO basis set and calculations. However, both approaches must produce close results for accurate calculations.

The COOP analysis for both CSF and KSF shows that only bonding Si–F orbitals (positive COOP values) are present below the Fermi level at 0 and 20 GPa (Figure 6). The charge density difference plot for the Si–F plane shows that electrons are localized at the Si–F bond (red regions), thus forming rigid directional covalent bonding (Figure 7a,c,e,g) independently of pressure. Also, the COOP of the Si–F bond is insensitive to the pressure, which is reflected in an unchanged bonding character of the Si–F bond at 0 and 20 GPa for the two materials. It is also in line with and explains the almost constant Si atomic charge as a function of pressure (Figure 5).

The COOP analysis of the A–F (A = Cs or K) bond demonstrates that, contrary to the Si–F case, both bonding and anti-bonding orbitals are now observed below the Fermi level (Figure 6). In particular, the anti-bonding orbitals are formed in the region close to the Fermi level, which is somewhat explained by a longer A–F bond length in comparison with the Si–F bond (Figure 3). These facts confirm the previous conclusion regarding the lower rigidity of the A–F bonds compared to the Si–F bonds. At 20 GPa, the anti-bonding A–F orbitals become more dominant, especially for CSF, as seen in Figure 6c,d. The bonding Cs–F orbitals, which are well seen for CSF at deeper energies, stem from a larger number of electrons and an involvement of Cs *d*-electrons in comparison with the K–F bond. The charge density difference plot for the A–F plane at 0 GPa shows spherical charge distribution around A- and F-ions, which is characteristic of ionic bonding (Figure 7b,d). On the one hand, at 20 GPa less charge is transferred from the A-ion to the F-ion than at 0 GPa (see the Mulliken charges listed in Table 2 and Figure 5). On the other hand, the electrons are localized in approximately 30% smaller volumes of the CsF_12_ and KF_12_ polyhedra due to the compressed crystal structure, which leads to an increase in the charge density on F- and A-ions (Figure 7f,h).

### 3.4. Vibrational and Dielectric Properties

By default, the CRYSTAL code performs calculations for a primitive unit cell, which for both CSF and KSF consists of *n* = 9 atoms. Therefore, both these systems have 3*n* = 27 normal lattice vibrations. Three of them are acoustic vibrations, and the others are optical vibrations. In both structures, theses 24 optical vibrations are distributed over 9 transverse optical (TO) vibrational modes. TO modes with their frequencies ν_0_, as calculated in this study for the CSF and KSF crystal, are presented in Table 4 and Table 5, respectively. The tables reveal that, for each crystal, the set of modes is identical: F_1g_ + 2F_2g_ + 3F_1u_ + F_2u_ + E_g_ + A_1g_, i.e., it includes seven triply degenerate modes (F-modes), one doubly degenerate mode (E_g_), and one non-degenerate mode (A_1g_). In both cases, three modes are IR-active (F_1u_ modes), four are Raman-active (2F_2g_ + E_g_ + A_1g_), and two are silent modes (F_1g_ and F_2u_), i.e., neither IR- nor Raman-active. Note that the IR- and Raman-active modes are strictly separated in both crystals (no one mode is both IR- and Raman-active), which is consistent with symmetry principles and the mutual exclusion rule for crystals with an inversion center. Naturally, the frequencies of modes in CSF and KSF are different. Firstly, it is notable that the F_2g_ and F_1g_ modes, which have the lowest frequencies, “exchange places” in the CSF and KSF systems. In CSF, the Raman-active F_2g_ mode has the lowest frequency (72 cm^−1^); while, in KSF, the silent F_1g_ mode is the lowest-frequency mode. In general, we conclude that the frequencies of TO modes in CSF are lower than in KSF, which is consistent with the presence of the heavier Cs atom in the CSF structure.

**Figure 8:**
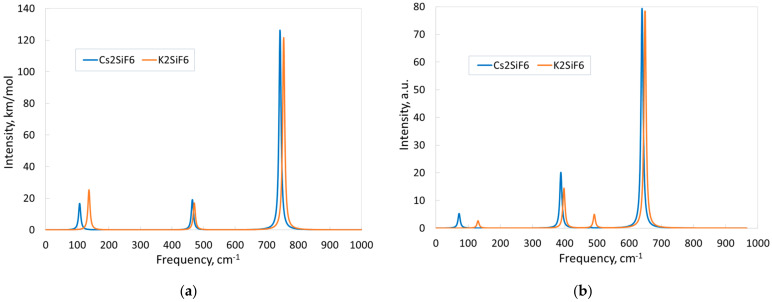
Simulated one-phonon IR and Raman spectra of CSF and KSF: (**a**) IR absorbance spectra; (**b**) Raman spectra.

The simulated IR and Raman spectra of CSF and KSF (Figure 8) confirm the latter conclusion: the peaks in the CSF spectra are slightly shifted to lower frequencies. Since all IR and Raman modes are well separated in frequency in the two crystals (Table 4 and Table 5), distinct individual peaks are observed in the spectra, each generated by only one vibrational mode.

Let us first consider the IR spectra (Figure 8a). Three IR-active F_1u_ modes form three peaks in each spectrum. An analysis of atom displacements in the IR-active modes leads to qualitatively identical conclusions for both materials. The dominant peak corresponds to the most high-frequency vibrational mode (742 cm^−1^ for CSF and 754 cm^−1^ for KSF). The Si atoms demonstrate maximum displacements in this mode and take part in vibrations along all axes. The fluorine atoms also contribute to this peak, but each of them vibrates mainly along only one axis. The A-atoms do not oscillate in this mode. The middle peaks (465 and 471 cm^−1^ for CSF and KSF, respectively) arise due to vibrations of F and Si atoms along all axes. Again, the A-atoms do not vibrate in these modes. Finally, the peaks, associated with the low-frequency IR-active mode (108 cm^−1^ for CSF and 137 cm^−1^ for KSF). All atoms vibrate in this mode along all axes. Note that the relatively heavy atoms (Cs and K) take part only in the lowest-frequency vibrations. However, some differences are observed in the character of vibrations of Cs and K atoms. In this mode, K atoms exhibit the largest displacements compared to the displacements of Si and F atoms in KSF. On the contrary, the displacements of Cs atoms are smaller than the displacements of Si and F atoms in CSF. This fact can be explained by the difference in the masses of the Cs and K atoms.

Now, we apply another method for analyzing the contribution of selected atoms to the vibrational mode—the isotopic substitution method, which is implemented in the CRYSTAL code [17] and makes it possible to modify the atomic masses of specific atoms. We compare the vibrational frequencies calculated with standard relative atomic masses with those obtained using the new, higher, isotopic masses. The idea is that an increase of atomic masses leads to a decrease of vibrational frequencies, but this decrease depends on a contribution of a corresponding atom (or group of atoms) to the particular vibrational mode. By comparing the vibrational modes frequency changes, the relative contributions of atoms to the different modes can be determined. We have used this technique previously in the study of KSF vibrational properties [15], and when investigating the point defects in diamonds [38,39]. In this study, we increased the relative masses of the CSF atoms by approximately 10%, and calculated the resulting isotopic shift in the frequencies of the vibrational modes. Calculations were performed for three different cases: (i) relative atomic masses of all Cs atoms were changed from 133 to 146; (ii) mass of Si atom was changed from 28 to 31; (iii) masses of all F atoms were changed from 19 to 21. The results of these calculations are collected in the last three columns of Table 4 (for CSF) and Table 5 (for KSF). In these tables, for example, the data in the Δ_Si_ column show the change in the frequencies (Δ = ν − ν_0_) of vibrational modes due to increasing the mass of the Si atom.

To begin, let us summarize the data presented in Table 4 and Table 5, focusing on the atoms composing the CSF and KSF crystals. The tables reveal that the Cs and K atoms vibrate only in two low-frequency vibrational modes, F_2g_ (Raman-active mode) and F_1u_ (IR-active mode). The Si atoms take part in the vibrations of three F_1u_ modes (all these modes are IR-active). Herewith, the contribution of the Si atoms to the highest-frequency mode is greater than to the other two modes. Finally, the F atoms oscillate in all modes except one Raman-active F_2g_ mode (72 cm^−1^ in CSF and 131 cm^−1^ in KSF) with the largest contribution to the Raman-active A_1g_ mode (640 cm^−1^ in CSF and 650 cm^−1^ in KSF).

Now, we return to the consideration of the IR-active modes. The isotopic shifts of the frequencies of specific modes fully confirm the estimates obtained from the atom displacement analysis. All atoms contribute to the first peak in the IR spectrum of CSF (associated with the vibrational mode of 108 cm^−1^), with the largest contribution from the F atoms. At the same time, of all the KSF atoms, the K atoms give the greatest contribution to the formation of the first peak in the IR spectrum. The second peak in the IR spectra of CSF and KSF arises due to vibrations of the Si and F atoms with the maximum contribution coming from the F atoms. The third (dominant) peak of the IR spectra is also contributed by the Si and F atoms, but the maximum contribution comes from the Si atom vibrations.

A few words about the silent modes from Table 4 and Table 5. The two silent modes in the CSF and KSF crystals are formed by vibrations of the F atoms only; the Si and K atoms remain stationary. The vibration of each F atom occurs in one plane.

Now, we discuss the Raman-active vibrational modes (Table 4 and Table 5). Like the silent modes, all Raman-active modes in the CSF and KSF crystals are exclusively associated with oscillations of the identical atoms: the low-frequency Raman mode (72 cm^−1^ for CSF and 131 cm^−1^ for KSF) with vibrations of the Cs or K atoms, and for the other three Raman modes, with F atom vibrations. An atom displacement analysis reveals that the Cs atoms vibrate along all axes in the 72 cm^−1^ mode. In the CSF crystal, the vibrations of each F atom in the 388 cm^−1^ mode occur in one plane, while in the 480 and 640 cm^−1^ modes, along one axis. The same conclusions are drawn for the corresponding atoms and modes in the KSF crystal (the vibrational properties of the KSF crystal were discussed in detail in our paper [15]).

Let us look at to the simulated Raman spectra (Figure 8b). In the case of the KSF crystal, four well-separated Raman modes generate four distinct peaks in the spectrum. In contrast to this, only three peaks are visible in the Raman spectrum of CSF, and it seems like the peak corresponding to the 480 cm^−1^ mode is missing. In fact, the peak is present, but it has very low intensity; the calculated intensity of the Raman line corresponding to the 480 cm^−1^ Raman-active mode is only 0.3% of the intensity of the line corresponding to the dominant peak in the spectrum.

It is interesting to compare the theoretically calculated Raman spectra (Figure 8b) with the experimental ones. The experimental Raman spectra of pure CSF and KSF measured at 300 K are available in Ref. [40]. Both experimental spectra consist of three peaks. Unfortunately, the spectra were measured in the frequency range from 200 to 700 cm^−1^. Therefore, it is not possible to discuss our calculated low-frequency peaks (72 cm^−1^ for CSF and 131 cm^−1^ for KSF). However, above 200 cm^−1^, the calculated spectra are in very good agreement with the experimental ones. In the experimental CSF spectrum, the peaks are located at the following frequencies: 407, 474, and 654 cm^−1^ (compared with the data for Raman-active modes in Table 4). The experimental spectrum of KSF exhibits peaks at slightly higher frequencies compared to the spectrum of CSF: 410, 480, and 658 cm^−1^. The calculated Raman spectra of KSF and CSF demonstrate the same trend (see Figure 8b, and Table 4 and Table 5). The deviation between the positions of the corresponding experimental and calculated peaks does not exceed 5% for CSF and is less than 3% for KSF. Moreover, the relative peak intensities in the experimental spectra qualitatively coincide with relative intensities of peaks in the simulated spectra. The most intensive peaks in all (experimental and theoretical) spectra fall in the region of ~650 cm^−1^; in addition, as stated in Ref. [40], the peaks in the region of ~480 cm^−1^ are very weak. Another experimental Raman spectrum of KSF–Mn^4+^ measured at ambient pressure is presented in Ref. [41]. This spectrum consists of four peaks at ~120, ~405, ~475, and ~655 cm^−1^, with a clearly dominant peak at ~655 cm^−1^. It is obvious that the experimental frequency values, which coincide very well with the calculated ones (Table 5), as well as the presence of a dominant peak at the corresponding frequency, indicate excellent agreement between the theoretical and experimental spectra. It should be noted that the authors of [40,41] claim that three high-frequency peaks in the Raman spectra are associated with vibrations of the SiF_6_ octahedra, which also coincides with the results of our analysis of atomic vibrations in the Raman-active modes of CSF and KSF.

Now, we briefly discuss the important dielectric parameter of system—the static dielectric tensor, which is closely related to the system vibrational properties. In the case of defect-free CSF and KSF, the static dielectric tensor is just a constant *ε*(0), since the three diagonal elements of the second rank tensor are equal, and the off-diagonal elements are zero. The static dielectric constant (static relative permittivity) *ε*(0) is the sum of electronic *ε_el_* and vibrational *ε_vib_*(0) components. The electronic (high-frequency) contribution *ε_el_* contains the electronic response, while the vibrational (ionic or lattice) contribution depends on the vibrational properties of the system, and is the sum of the vibrational contributions of all IR-active modes. Our calculations given for the electronic component, also called the optical dielectric constant (optical relative permittivity), the value *ε_el_* = 1.882 for CSF and *ε_el_* = 1.745 for KSF. Note that the square root of these values gives the refractive index value presented in Table 1, which is in very good agreement with the experimental data. The three IR-active modes of CSF and KSF contribute to the vibrational component *ε_vib_*(0) of the static dielectric constant *ε*(0). Our calculations reveal that, for CSF, *ε_vib_*(0) = 2.162. Wherein, the main contribution to the *ε_vib_*(0) (1.775 from 2.162) comes from the vibrational mode at 108 cm^−1^—the IR-active vibrational mode with the lowest frequency. This is expected, since the contributions of vibrational modes to *ε_vib_*(0) is inversely proportional to the square of the frequency [15]. Thus, we calculated that the static dielectric constant of CSF is *ε*(0) = 4.044. For KSF *ε_vib_*(0) = 2.628, again with the main contribution (2.160 from 2.628) from the IR-active vibrational mode with the lowest frequency (137 cm^−1^). The calculated static dielectric constant of KSF is 4.373. To summarize, we can say that the considered dielectric properties of CSF and KSF are quite close; however the vibrational contribution to the static dielectric constant is slightly larger for the KSF crystal.

### 3.5. Debye Temperature Evaluation

In this subsection, the dependence of elastic wave (sound) velocities and the Debye temperature in the CSF crystal on external pressure will be estimated and compared with similar dependences for the KSF crystal. The corresponding calculations for KSF were performed in Refs. [10,15]; the authors have no information about such calculations for the CSF crystal.

The longitudinal *v_l_* and transverse *v_t_* sound velocities in crystal can be calculated using the following equation [10,13,34]:

(3) $$v_{l}=\sqrt{\frac{3B+4G}{3\rho }}\;,\;\;\;\;\;\;\;v_{t}=\sqrt{\frac{G}{\rho }\;,}$$

where *B* is the bulk modulus, *G* is the shear modulus, and *ρ* is the density of the material. Note that the density included in Formula (3) also depends on pressure and, at each pressure, its value for the optimized structure was calculated. The average elastic wave velocity (an effective sonic velocity) *v_m_*, weighted by the number of polarization states, can be calculated using *v_l_* and *v_t_* as follows [10,13,34]:

(4) $$v_{m}={\left[\frac{1}{3}\left(\frac{2}{v_{t}^{3}}+\frac{1}{v_{l}^{3}}\right)\right]}^{-1/3}.$$

The Debye temperature *Θ_D_* is proportional to *v_m_* and is calculated by the following formula [10,13,34]:

(5) $$\Theta_{D}=\frac{h}{k}{\left(\frac{3nN_{A}}{4\pi }\frac{\rho }{\mu }\right)}^{1/3}v_{m}\;,$$

where *h* and *k* are the Planck’s and Boltzmann’s constants, respectively, *N_A_* is the Avogadro’s number, *μ* is the molecular weight, and *n* denotes the number of atoms per formula unit (9 for CSF and KSF).

Thus, using the *B* and *G_H_* moduli from Table 3 and Equations (3)–(5), the sound velocities *v_l_*, *v_t_*, and *v_m_*, as well as the Debye temperature *Θ_D_*, can by calculated as functions of the external pressure. The results of these calculations, along with the pressure-dependent CSF density, are collected in Table 6.

First, let us compare the densities of the CSF and KSF crystals. At the ambient pressure, the CSF density is 3887 kg/m^3^ (see Table 6), which is significantly higher than the density of the KSF (2763 kg/m^3^ [15]). The densities of both crystals increase monotonically with pressure, and at 20 GPa the increase of the CSF density is ~44%, while the increase of the KSF density is ~40%. From this fact we can once again conclude that the rigidity of the KSF crystal is slightly higher than that of the CSF.

**Figure 9:**
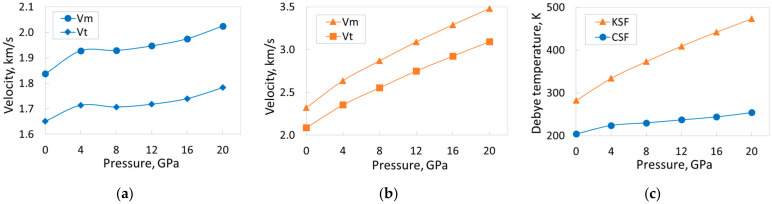
Calculated dependences of transverse *v_t_* and effective (mean) *v_m_* sound velocities, as well as Debye temperature, on pressure: (**a**) sound velocities in CSF; (**b**) sound velocities in KSF; (**c**) Debye temperatures. The data for KSF are taken from Ref. [15]. The lines in the figures are shown only as a guide to the eyes.

As was shown in Ref. [15], all sound velocities (*v_l_*, *v_t_*, *v_m_*) and the Debye temperature increase monotonically with increasing pressure in KSF. The authors of Ref. [10] have drawn the same conclusion for the KSF crystal. Table 6 reveals that the situation with the CSF crystal is a little more complicated. Namely, the monotonous increase in transverse velocity *v_t_* between 4 GPa and 12 GPa is violated. Probably, there is also a local minimum in the specified pressure range for the *v_m_* velocity. As a result, when the pressure increases from 0 to 20 GPa, the increase in velocity *v_t_* is about 8%, and *v_m_* is about 10% (Figure 9a). For comparison, these values for the KSF crystal are 48% and 50%, respectively [15] (see Figure 9b). Note that, in the range of 0–20 GPa, velocities *v_l_*, *v_t_*, and *v_m_*, as well as the Debye temperature, are higher for the KSF crystal. The growth rate of all these parameters is also higher for the KSF crystal. The velocity *v_l_* increases by 67% for CSF (change of pressure 0–20 GPa), and by 77% for KSF; the Debye temperature increases by 25% and 68% for the CSF and KSF crystals, respectively (Figure 9c). The lack of both experimental data and other calculations does not allow us to present any comparison of the obtained results.

## 4. Conclusions

In this paper, the CRYSTAL23 computer code within the LCAO method of the density functional theory, employing the advanced hybrid exchange-correlation B1WC functional, was used for the first-principle simulations of a wide range of structural and physico-chemical properties of perfect CSF and KSF crystals. The use of the hybrid functional and Gaussian basis sets for the study of these materials is a distinctive feature of this study. The structural, electronic, and elastic properties of compounds were considered and compared in detail; obtained results were discussed and compared with available experimental data and with the results of other calculations. Special attention was paid to the electronic properties of the materials. In particular, the crystalline orbital overlap population (COOP) analysis was used to determine the nature of the Si–F, Cs–F, and K–F bonds. The vibrational and dielectric properties of CSF and KSF were calculated, and one-phonon IR and Raman spectra were simulated. The effect of hydrostatic pressure (in the range of 0–20 GPa) on selected structural, the electronic and elastic properties, as well as on the Debye temperature, was analyzed.

Summing up, our calculations reveal that both the CSF and KSF crystals are wide-gap insulators, and the band gaps of both crystals increase monotonically with pressure, but the rate of increase is different. The obtained results demonstrate that the rigidity of the Si–F bond in both crystals is significantly higher than that of the Cs–F or K–F bonds. Moreover, the decrease in the volume of the unit cell of CSF and KSF with pressure occurs mainly due to the decrease in the size of the CsF_12_ and KF_12_ polyhedra, respectively. The COOP analysis for CSF and KSF shows that, below the Fermi level, only bonding Si–F orbitals are observed, whereas for the A–F bonds both bonding and anti-bonding orbitals are present, which may explain the differences in bond rigidity. Interestingly, the charge of the Si-ion remains almost unchanged under external pressure in both crystals, while the charges (in absolute value) of the A-cation and F-anion decrease monotonically with increasing pressure. This may indicate that the ionicity of the A–F bonds slightly decreases. Analysis of the influence of external pressure on the elastic properties of both materials shows that all considered elastic parameters increase with increasing pressure, however the rate of increase can be very different. In general, the calculations reveal that the KSF crystal is more rigid than the CSF crystal. The calculations also revealed differences in the dependence of sound speeds on pressure in the CSF and KSF crystals. The method of isotopic substitution was used for the analysis of vibrational properties of the crystals, as well as the IR and Raman spectra. The Raman spectra of CSF and KSF demonstrated excellent agreement with the experimental data.

The comprehensive data set obtained in this study provides valuable reference information on the properties of the CSF and KSF crystals, and will be used in the future modeling of the luminescent properties of various impurities (e.g., Mn^4+^) in these materials.

## Figures and Tables

**Table 1:** Calculated lattice constant *a*, dimensionless free *x* coordinate of the F ion (Wyckoff position 24e), interionic distances (Si–F, A–F), band gap *E_g_*, Mulliken charges of ions *q*, and refractive index *n* of CSF and KSF at 0 GPa, along with the corresponding available experimental data (values in the parenthesis).

	Cs_2_SiF_6_	K_2_SiF_6_
*a*, Å	8.866 (8.890 [12])	8.086 (8.134 [13], 8.046 [24])
*x*	0.1919	0.2100 (0.2095 [24])
Si–F, Å	1.702 (1.695 [12])	1.698 (1.683 [13])
A–F, Å	3.176	2.877 (2.897 [13])
*E_g_*, eV	10.39	9.73
*q*(A) (Cs or K), |e|	0.922	0.852
*q*(Si), |e|	1.809	1.807
*q*(F), |e|	−0.609	−0.585
*n*	1.37 (1.38–1.39 [25])	1.32 (1.34 [13])

**Table 2:** Mulliken (effective atomic) charges (in |e|) of ions depending on the external pressure.

Pressure, GPa	Cs_2_SiF_6_			K_2_SiF_6_		
	Cs	Si	F	K	Si	F
0	0.922	1.809	−0.609	0.852	1.807	−0.585
4	0.899	1.801	−0.600	0.824	1.802	−0.575
8	0.880	1.798	−0.593	0.807	1.803	−0.569
12	0.861	1.796	−0.586	0.793	1.807	−0.566
16	0.843	1.797	−0.580	0.783	1.811	−0.563
20	0.827	1.799	−0.575	0.774	1.817	−0.561

**Table 3:** Effect of pressure on the elastic constants (*C*_11_, *C*_12_, *C*_44_), bulk modulus *B*, Hill shear modulus *G_H_*, Hill Young’s modulus *E_H_* (all in GPa), and dimensionless Hill Poisson’s ratio *ν_H_* of CSF.

Pressure, GPa	*C*_11_	*C*_12_	*C*_44_	*B*	*G_H_*	*E_H_*	*ν_H_*
0	33.10	12.48	10.80	19.35	10.60	26.89	0.268
4	55.48	33.60	14.78	40.90	13.10	35.52	0.355
8	73.83	51.56	16.45	58.98	14.07	39.10	0.390
12	91.70	68.62	18.14	76.31	15.13	42.58	0.407
16	109.66	85.55	19.92	93.59	16.29	46.18	0.418
20	128.12	102.40	22.09	110.98	17.78	50.64	0.424

**Table 4:** Calculated frequencies ν_0_ of transverse optical (TO) vibrational modes at the Γ-point of the CSF Brillouin zone (BZ). Calculated isotopic shift of frequencies (Δ_Cs_, Δ_Si_, Δ_F_) after increasing the relative masses of the corresponding atoms (Cs, Si, or F) in CSF by 10%. Letters A and I in the third column indicate whether the mode is, respectively, active (A) or inactive (I) for IR and Raman scatterings.

Mode	Frequency ν_0_, cm^−1^	IR–Raman Activity	Cs Isotopic Shift Δ_Cs_, cm^−1^	Si Isotopic Shift Δ_Si_, cm^−1^	F Isotopic Shift Δ_F_, cm^−1^
F_2g_	72.0	I–A	−3.3	0.0	0.0
F_1g_	104.2	I–I	0.0	0.0	−5.1
F_1u_	107.5	A–I	−1.7	−0.7	−2.8
F_2u_	265.1	I–I	0.0	0.0	−12.9
F_2g_	388.0	I–A	0.0	0.0	−18.9
F_1u_	464.5	A–I	0.0	−4.2	−19.2
E_g_	480.4	I–A	0.0	0.0	−23.5
A_1g_ *	640.2	I–A	0.0	0.0	−31.2
F_1u_	742.3	A–I	0.0	−23.4	−12.6

* Note that this mode is denoted as A_g_ in the CRYSTAL output file.

**Table 5:** Calculated frequencies ν_0_ of transverse optical (TO) vibrational modes at the Γ-point of the KSF Brillouin zone (BZ). Calculated isotopic shift of frequencies (Δ_K_, Δ_Si_, Δ_F_) after increasing the relative masses of the corresponding atoms (K, Si, or F) in KSF by 10%. Letters A and I in the third column indicate whether the mode is, respectively, active (A) or inactive (I) for IR and Raman scatterings.

Modes	Frequency ν_0_, cm^−1^	IR–Raman Activity	K Isotopic Shift Δ_K_, cm^−1^	Si Isotopic Shift Δ_Si_, cm^−1^	F Isotopic Shift Δ_F_, cm^−1^
F_1g_	71.9	I–I	0.0	0.0	−3.5
F_2g_	130.8	I–A	−6.3	0.0	0.0
F_1u_	137.0	A–I	−4.2	−0.4	−2.0
F_2u_	263.6	I–I	0.0	0.0	−12.9
F_2g_	397.6	I–A	0.0	0.0	−19.4
F_1u_	470.5	A–I	0.0	−4.6	−19.1
E_g_	491.8	I–A	0.0	0.0	−24.0
A_1g_ *	649.5	I–A	0.0	0.0	−31.7
F_1u_	753.7	A–I	0.0	−23.2	−13.2

* Note that this mode is denoted as A_g_ in the CRYSTAL output file.

**Table 6:** Dependences of CSF density *ρ* (kg/m^3^), the sound velocities *v_l_*, *v_t_*, *v_m_* (m/s) in crystal and Debye temperature *Θ_D_* (K) on pressure. The data in this table were calculated using results of first-principle simulations.

Pressure, GPa	*ρ*	*v_l_*	*v_t_*	*v_m_*	*Θ_D_*
0	3887	2935	1651	1837	204
4	4459	3618	1714	1928	224
8	4831	4011	1707	1929	230
12	5127	4338	1718	1947	237
16	5378	4630	1740	1975	244
20	5593	4907	1783	2025	254

## Data Availability

The original contributions presented in this study are included in the article, further inquiries can be directed to the corresponding authors.
